# Arginine‐Rich Cell‐Penetrating Peptides Induce Lipid Rearrangements for Their Active Translocation across Laterally Heterogeneous Membranes

**DOI:** 10.1002/advs.202404563

**Published:** 2024-06-26

**Authors:** Sujin Park, Jinmin Kim, Seung Soo Oh, Siyoung Q. Choi

**Affiliations:** ^1^ Department of Chemical and Biomolecular Engineering Korea Advanced Institute of Science and Technology (KAIST) Daejeon 34141 Republic of Korea; ^2^ Department of Materials Science and Engineering Pohang University of Science and Technology (POSTECH) Pohang 37673 Republic of Korea; ^3^ Institute for Convergence Research and Education in Advanced Technology (I‐CREATE) Yonsei University Incheon 21983 Republic of Korea

**Keywords:** cell‐penetrating peptide, freestanding lipid bilayer, giant unilamellar vesicle, membrane remodeling, real‐time visualization

## Abstract

Arginine‐rich cell‐penetrating peptides (CPPs) have emerged as valuable tools for the intracellular delivery of bioactive molecules, but their membrane perturbation during cell penetration is not fully understood. Here, nona‐arginine (R_9_)‐mediated membrane reorganization that facilitates the translocation of peptides across laterally heterogeneous membranes is directly visualized. The electrostatic binding of cationic R_9_ to anionic phosphatidylserine (PS)‐enriched domains on a freestanding lipid bilayer induces lateral lipid rearrangements; in particular, in real‐time it is observed that R_9_ fluidizes PS‐rich liquid‐ordered (L_o_) domains into liquid‐disordered (L_d_) domains, resulting in the membrane permeabilization. The experiments with giant unilamellar vesicles (GUVs) confirm the preferential translocation of R_9_ through L_d_ domains without pore formation, even when L_o_ domains are more negatively charged. Indeed, whenever R_9_ comes into contact with negatively charged L_o_ domains, it dissolves the L_o_ domains first, promoting translocation across phase‐separated membranes. Collectively, the findings imply that arginine‐rich CPPs modulate lateral membrane heterogeneity, including membrane fluidization, as one of the fundamental processes for their effective cell penetration across densely packed lipid bilayers.

## Graphical abstract

This work presents a real‐time visualization of dynamic membrane reorganization during the binding and penetration of arginine‐rich cell‐penetrating peptides. The peptides induce a phase transition in laterally heterogeneous membranes, leading to membrane fluidization. This modulation of lateral membrane structure actively facilitates the translocation of arginine‐rich cell‐penetrating peptides across the cell membranes.

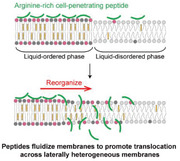

## 1 Introduction

The transport of active molecules (e.g., ions, metabolites, and drugs) across cell membranes has been significantly challenging due to their limited diffusion through phospholipid bilayers. To address this, cell‐penetrating peptides (CPPs) have attracted great attention because of their ability to cross cell membranes with cargoes.^[^ ^1^, ^2^ ^]^ In particular, arginine‐rich peptides, including HIV‐1 TAT peptide and polyarginines (R_n_), are widely used for imaging and therapeutic applications.^[^ ^3^ ^]^ While energy‐dependent endocytosis has been regarded as a major route of cellular internalization of various CPPs, growing evidence reveals that arginine‐rich CPPs could be directly translocated across membranes in an energy‐independent manner.^[^ ^4^, ^5^, ^6^, ^7^, ^8^ ^]^ The direct translocation of arginine‐rich CPPs has been suggested to be related to their strong interactions with lipid headgroups. Specifically, the positively charged arginine prefers to bind to negatively charged lipids (e.g., phosphatidylserine (PS) and phosphatidylglycerol (PG)), and the guanidinium group of arginine even forms hydrogen bonds with phosphate, carboxylate or sulfate groups of lipids, causing structural alterations of peptide‐bound membranes.^[^ ^9^, ^10^ ^]^

Multiple mechanisms have been proposed to explain how CPPs traverse cell membranes. One mechanism is that the binding of CPPs induces stable pores in lipid membranes for subsequent cell penetration of molecules. The strong interactions between arginine‐rich CPPs and lipids are well known to significantly reduce the thermodynamic cost of membrane pore formation; both HIV‐1 TAT^[^ ^11^, ^12^, ^13^ ^]^ and R_n_^[^ ^14^, ^15^, ^16^ ^]^ have proven to induce pores in membranes, allowing the passage of small dyes or ions. The other mechanism suggests that when transient pores naturally occur with thermal fluctuations, CPPs utilize these pores in translocation.^[^ ^15^, ^17^ ^]^ Based on the classical theory of pore formation,^[^ ^18^ ^]^ the probability of both stable and transient pore formation is mainly determined by two factors; the lateral tension of the membrane, which promotes pore expansion, and the line tension of the pore edge, which promotes pore closure. Accordingly, the translocation ability of CPPs has been found to be influenced by the mechanical properties of lipid membranes, relevant to lipid chain length and saturation, and cholesterol levels.^[^ ^19^, ^20^, ^21^, ^22^ ^]^

Another fundamental feature of cellular membranes is their lateral heterogeneity.^[^ ^23^, ^24^ ^]^ Several studies have shown that the alterations in lateral membrane organization have a significant role in the functionality of diverse membrane‐active peptides. Antimicrobial peptides (AMPs) have been suggested to disrupt bacterial biological functions by affecting the lateral mobility of membrane components and the distribution of membrane proteins.^[^ ^25^, ^26^, ^27^, ^28^ ^]^ Phase separation induced by a few CPPs has also been reported, raising the possibility of peptide translocation via phase boundary defects^[^ ^29^, ^30^, ^31^ ^]^ or modulated membrane curvatures.^[^ ^32^ ^]^ While it is evident that the translocation ability of the CPPs could not be fully understood without membrane heterogeneity, the relationship between CPP translocation and dynamic membrane rearrangements in heterogeneous membranes remains poorly understood. Instead, penetration efficiencies have typically been evaluated by externally modulating overall membrane properties by changing membrane compositions.^[^ ^7^, ^12^, ^15^, ^33^, ^34^ ^]^

In this work, we demonstrate the dissolution of ordered lipid domains in heterogeneous membranes upon the binding of nona‐arginine (R_9_), a representative arginine‐rich CPP,^[^ ^35^, ^36^ ^]^ that can facilitate peptide translocation across the membrane. Using planar freestanding lipid membranes,^[^ ^37^ ^]^ we successfully visualized the lateral rearrangements induced by R_9_ in real‐time. In particular, we found that the lipid rearrangements could lead the liquid‐ordered (L_o_) phase to fluidize into the liquid‐disordered (L_d_) phase, thereby lowering the phase transition temperature of the lipid bilayers. Penetration analysis of R_9_ into phase‐separated giant unilamellar vesicles (GUVs) further confirmed that the dissolving of the L_o_ domains accelerates the direct translocation of R_9_. These findings suggest that peptide‐induced membrane remodeling may accelerate membrane translocation of CPPs by altering the fluidity of tightly packed lipid membranes.

## 2 Results

### 2.1 Experimental Setup for Real‐Time Monitoring of Lateral Membrane Reorganization

**Figure 1:**
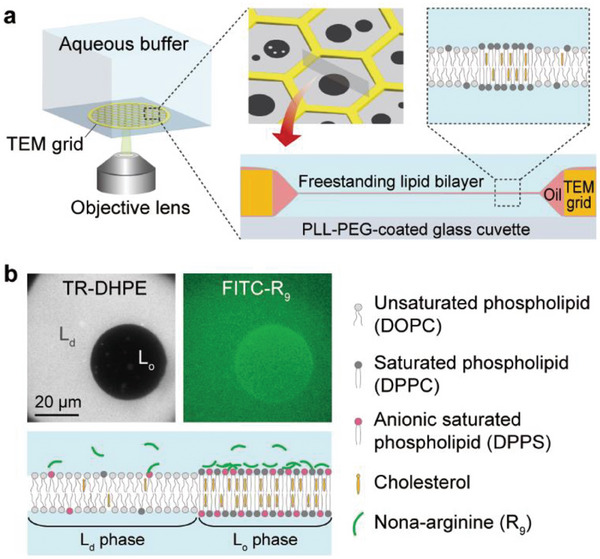
Freestanding planar lipid membrane array for analyzing R_9_‐induced membrane reorganization. a) Experimental setup for real‐time monitoring of lateral membrane reorganization. Multiple lipid membranes are created in hexagonal holes of a transmission electron microscopy (TEM) grid when immersed in an aqueous buffer. The membrane array is observed in real‐time through an inverted fluorescence microscope. The detailed experimental procedure is illustrated in Figure S1 (Supporting Information). b) Phase‐separated lipid membrane containing anionic saturated phospholipid, DPPS. The lipid membrane composed of unsaturated phospholipids (DOPC), saturated phospholipids (DPPC), and cholesterol undergoes phase separation into a liquid‐disordered (L_d_) phase (bright) and liquid‐ordered (L_o_) phase (dark) at 24 °C. When cationic nona‐arginine (R_9_) is introduced, it electrostatically binds to the surface of lipid membranes, specifically interacting with anionic phospholipids. The heterogeneous membrane is formed using a lipid oil mixture of DOPC/DPPC/DPPS + TR‐DHPE (60/30/10 + 0.5 mol%), and cholesterol is added via MβCD. The prepared FITC‐R_9_ solution was injected at a concentration of 3 µm into the aqueous solution above the lipid membranes. The images were obtained ≈1 min after the FITC‐R_9_ addition.

To visualize the dynamic membrane reorganization induced by nona‐arginine (R_9_), we used a freestanding planar lipid membrane array in a transmission electron microscopy (TEM) grid (**Figure** **1**a; Figure S1, Supporting Information).^[^ ^37^, ^38^ ^]^ We prepared simplified lipid mixtures composed of phosphatidylcholine (PC), phosphatidylserine (PS), and cholesterol, mimicking mammalian plasma membranes. The preparation of phase‐separated membranes began with the mixing of unsaturated phospholipid (DOPC) and saturated phospholipid (DPPC), both of which are neutral in charge, and the resulting DOPC/DPPC/cholesterol membrane underwent phase separation into a bright L_d_ phase and a dark L_o_ phase at 24 °C with 0.5 mol% of Texas Red 1,2‐dihexadecanoyl‐sn‐glycero‐3‐phosphoethanolamine (TR‐DHPE) (Figure 1b). We note that the L_d_ phase is enriched in unsaturated phospholipids and fluorescent TR‐DHPE, and the L_o_ phase is enriched in saturated phospholipids and cholesterol. Either the L_d_ or L_o_ phase can be more negatively charged to strongly interact with the positively charged R_9_; by introducing PS, an anionic phospholipid, in a saturated or unsaturated form (i.e., DPPS or DOPS), we could determine which phase preferentially interacted with the cationic peptide. For example, upon the inclusion of DPPS, the fluorescence of FITC‐labeled R_9_ (FITC‐R_9_) was predominately found in the L_o_ phase, where anionic saturated phospholipids were more abundant (Figure 1b). Even with GUVs, we successfully visualized the phase‐preferential R_9_ binding (Figure S2, Supporting Information), and the R_9_ preferred binding to the L_d_ or L_o_ phase with 10 mol% DOPS or DPPS, respectively, whereas the binding did not occur on the neutral membrane of DOPC/DPPC/cholesterol.

### 2.2 R_9_‐Induced Lipid Rearrangements on Phase‐Separated Membranes

**Figure 2:**
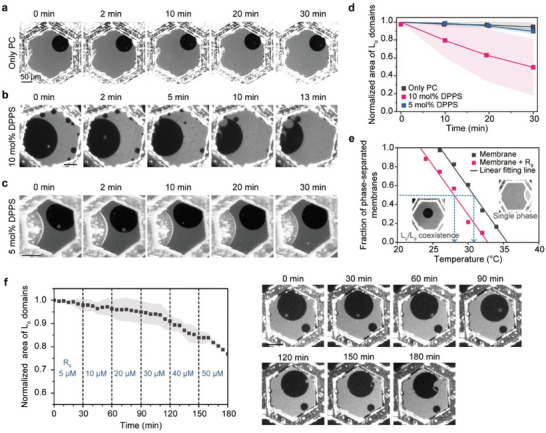
R_9_‐induced membrane reorganization on heterogeneous membranes. a–c) Membrane dynamics with different lipid compositions upon addition of 50 µm R_9_. No change in phase behavior was observed with neutral PC membranes a), whereas both phase separation and mixing were found with anionic DPPS‐containing, phase‐separated membranes (b‐c). d) Normalized area of L_o_ domains over time. The normalized area (*A_R_*) is defined as *A_R_ = A/A_0_*, where *A* and *A_0_* indicate the area of L_o_ domains at each time point and the initial area of L_o_ domains before the addition of R_9_. Data represent mean ± SD (*n* = 4–11). e) Fraction of phase‐separated membranes as a function of temperature. The apparent miscibility transition temperature (T_m_) was determined for bare DOPC/DPPC/DPPS/cholesterol membranes (30.1 °C) and after 30 min incubation with 50 µm R_9_ (28.2 °C) by fitting the counted fractions to a linear function. Data were obtained from n = 40–92 planar membranes. f) Left: Normalized area of L_o_ domains over time. R_9_ concentrations increased from 5 to 50 µm every 30 min. Data represent mean ± SD (n = 2). Right: Phase‐separated membranes at each R_9_ concentration at each time. L_o_ domain dissolution was accelerated with increasing R_9_ concentrations. The membranes were formed using a lipid oil mixture of DOPC/DPPC + TR‐DHPE (60/40 + 0.5 mol%) a), DOPC/DPPC/DPPS + TR‐DHPE (60/30/10 + 0.5 mol%) b–f), DOPC/DPPC/DPPS + TR‐DHPE (75/20/5 + 0.5 mol%) c), and cholesterol was added via MβCD. Imaging was performed at 24 °C. Scale bars, 50 µm.

On heterogeneous membranes, the binding of cationic R_9_ induced lateral rearrangements of anionic lipids (**Figure** **2**). The phase behaviors of freestanding lipid bilayers were monitored in real‐time by introducing 50 µm R_9_ to the membranes while varying the membrane compositions. On neutral DOPC/DPPC/cholesterol membranes, the addition of positively charged R_9_ had no effect due to the absence of negatively charged lipids (Figure 2a). However, when cationic DPPS was included in the heterogeneous membranes, two significant changes in phase behavior occurred (Figure 2b; Movie S1, Supporting Information). First, immediately upon the addition of R_9_, small L_o_ domains were formed within the pre‐existing L_d_ domains as a result of R_9_ binding (Figure S3, Supporting Information). It is known that electrostatic interactions between anionic lipids and cationic peptides can induce lateral segregation of anionic lipids,^[^ ^28^, ^30^, ^39^, ^40^ ^]^ and we also confirmed that initially homogeneous DOPC/DPPS membranes underwent phase separation due to the DPPS clustering upon the addition of R_9_ (Figure S3, Supporting Information), making FITC‐R_9_ highly concentrated on the surface of newly formed L_o_ domains (Figure S4, Supporting Information). Second, over several tens of minutes, the total area of L_o_ domains decreased dramatically as a result of domain dissolution, that is, phase mixing. On membranes containing 10 mol% DPPS, for example, L_o_ domains decreased to 49.5 ± 31.4% (n = 4) of the initial area after 30 min (Figure 2d). Similarly, with 4 µm of HIV‐1 TAT, another representative arginine‐rich CPP, the L_o_ domains of membranes containing 16 mol% DMPG were reduced to 53.7 ± 7.1% (n = 10) of the initial area after 1 h (Figure S5, Supporting Information). We note that there were no alterations in the phase behavior with membranes containing DOPS (Figure S6, Supporting Information). It was anticipated that these membranes would undergo lateral rearrangements in a similar manner; however, the inability of unsaturated lipid DOPS to form dark ordered phase domains made their observation technically challenging through domain visualization based on dye partitioning.^[^ ^41^, ^42^ ^]^

Despite the early temporal clustering of DPPS‐rich ordered domains within the L_d_ phase, DPPS‐rich L_o_ domains, including newly formed ones, eventually dissolved over time. The observed phase mixing suggests that the binding of cationic R_9_ to anionic L_o_ domains would ultimately decrease the miscibility temperature (T_m_) of lipid phase separation. To analyze the temperature‐dependent phase separation behavior of laterally heterogeneous membranes, the temperature of an imaging chamber was increased at a rate of ≈0.4 °C min^−1^, and the number of phase‐separated membranes was counted every 2 °C. By linearly fitting the collected data, the apparent T_m_ was determined as the temperature at which 50% of the total membranes undergo phase separation (Figure 2e). For example, the T_m_ of bare DOPC/DPPC/DPPS/cholesterol membranes was measured to be 30.1 °C, but 30 min incubation with 50 µm R_9_ lowered the T_m_ to 28.2 °C. This decrease in T_m_ indicates that the cationic R_9_ fluidizes the anionic lipid membranes, leading to the observed L_o_ domain dissolution by phase mixing.

### 2.3 Electrostatic Interactions of R_9_ with Heterogeneous Membranes in Phase Mixing

As the dissolution of L_o_ domains is likely due to the electrostatic interactions between the cationic R_9_ and the anionic DPPS in the DPPS‐containing heterogeneous membranes, we next investigated the effect of DPPS and R_9_ concentration, respectively. We first varied the molar content of DPPS in the phase‐separated membranes, and the total area of L_o_ domains was measured over time (Figure 2b–d). While 10 mol% DPPS resulted in a decrease of ≈50.5% in the area of the L_o_ domains (Figure 2b), 5 mol% DPPS did not cause such a significant change (Figure 2c). It should be noted that we could not investigate the effect of higher mol% DPPS; as the percentage of DPPS increased, the resulting lipid bilayer became increasingly unstable, impeding the formation of freestanding planar lipid membranes in a TEM grid. However, the effect of R_9_ concentrations on the extent of L_o_ domain dissolution was well evaluated when the DPPS content was up to 10 mol%. When the R_9_ concentration was gradually changed from 5 to 50 µm for 180 min, the decrease in the total area of L_o_ domains became more significant at higher R_9_ concentrations (Figure 2f). Although we only observed the membranes for 30 min at each concentration, phase mixing occurred slowly and continuously even at low R_9_ concentrations, and were able to identify the trend of R_9_ concentration‐dependent dissolution of the L_o_ domains. Note that in our experiment system, the lipid‐to‐peptide ratio is expected to be ≈1:10 at 50 µm R_9_, potentially leading to dramatic phase mixing at this high peptide concentration.

The real‐time observations indicated that the rate of phase mixing would be accelerated with higher levels of anionic lipids and cationic peptides. When there were stronger interactions between the anionic lipids and the cationic peptides, the T_m_ of phase‐separated membranes would decrease further, leading to more domain dissolution by phase mixing. Indeed, the dissolution of L_o_ domains on heterogeneous membranes became faster with increasing concentrations of anionic DPPS and cationic R_9_ (Figure 2d–f); even at low concentrations of DPPS and R_9_, the membrane reorganization would occur, although with a limited extent of phase mixing. Thus, we concluded that the presence of R_9_ would induce membrane reorganization and subsequent fluidization, allowing us to speculate that arginine‐rich CPP‐mediated lipid rearrangements would be directly related to its active translocation across laterally heterogeneous membranes.

### 2.4 Investigating Penetration of R_9_ into Homogeneous and Heterogeneous GUVs

**Figure 3:**
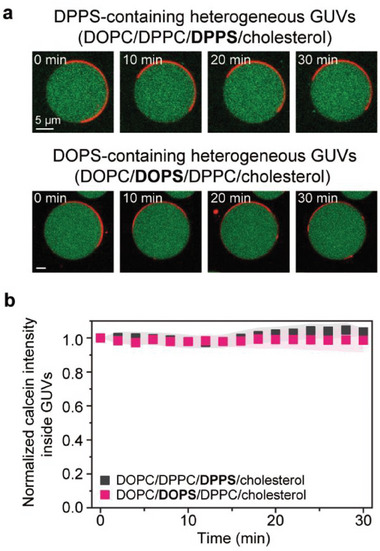
Calcein leakage assay to confirm pore formation. a) Calcein‐encapsulated DOPC/DPPC/DPPS/cholesterol GUVs (top) and DOPC/DOPS/DPPC/cholesterol GUVs (bottom) over time after the addition of 5 µm R_9_. Red and green represent TR‐DHPE and calcein, respectively. b) Normalized calcein fluorescence intensity inside the GUVs over time. Data represent mean ± SD (n = 23–35). Membrane composition: DOPC/DPPC/DPPS/cholesterol/biotin‐cap‐PE + TR‐DHPE (33/25/10/30/2 + 0.2 mol%) and DOPC/DOPS/DPPC/cholesterol/biotin‐cap‐PE + TR‐DHPE (23/10/35/30/2 + 0.2 mol%). Imaging was performed at 20 °C. Scale bars, 5 µm.

The energy‐independent cell penetration of arginine‐rich CPPs is generally explained by the aforementioned two mechanisms, with the critical difference being whether or not the peptide is directly involved in pore formation at plasma membranes. Using PS‐containing heterogeneous membranes, we explored the possibility of R_9_‐induced pore formation and its membrane penetration. To evaluate the pore‐forming ability of R_9_, we encapsulated calcein, a water‐soluble fluorescent dye (≈6.5 Å in radius), into DOPC/DPPC/DPPS/cholesterol or DOPC/DOPS/DPPC/cholesterol GUVs, and observed the dye leakage from the interior to the exterior of the GUVs in real‐time (**Figure** **3**). Because the GUVs were unstable at high R_9_ concentrations (>50 µm), our experimental setup was limited to 10 µm R_9_, making our leakage measurements reliable and reproducible. It should be noted that this concentration of peptide was still capable of inducing a phase transition from L_o_ to L_d_ phase. Over 30 min, the addition of R_9_ did not cause notable fluorescence changes inside the GUVs (Figure 3a), indicating that there were no large pores for the calcein to pass through. Moreover, there was no big difference in dye leakage between DPPS‐ and DOPS‐containing membranes, confirming that phase‐preferential R_9_ binding would be irrelevant to pore formation as well (Figure 3b).

**Figure 4:**
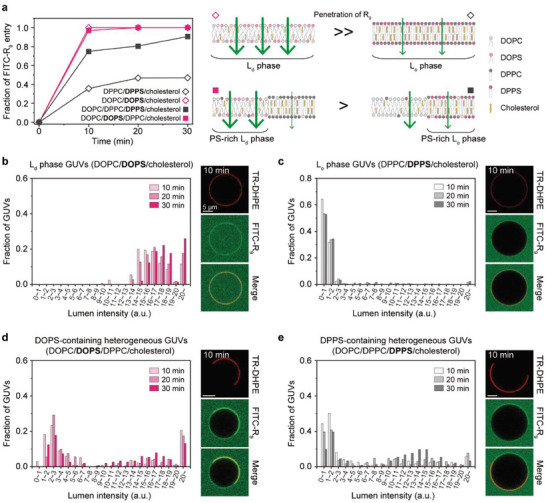
Phase‐dependent translocation of FITC‐R_9_ into GUVs. a) Left: Fraction of FITC‐R_9_ entry into the GUVs of four different compositions over time. 5 µm FITC‐R_9_ was applied. Right: Comparison of phase‐dependent R_9_ entry into homogeneous GUVs (top) and heterogeneous GUVs (bottom). b–e) Histograms for the distribution of lumen intensities over time for each composition of GUVs. Confocal fluorescence microscopy images of GUVs after 10 min incubation with 5 µm FITC‐R_9_ are at the right side of each histogram. Two different single‐phase GUVs were analyzed: L_d_ phase GUVs composed of DOPC/DOPS/cholesterol (empty red diamond in a,b) and L_o_ phase GUVs composed of DPPC/DPPS/cholesterol (empty black diamond in a,c). Two different phase‐separated GUVs were analyzed: DOPC/DOPS/DPPC/cholesterol GUVs with a PS‐rich L_d_ phase (solid red square in a, d) and DOPC/DPPC/DPPS/cholesterol GUVs with a PS‐rich L_o_ phase (solid black square in a,e). Membrane composition: DOPC/DOPS/cholesterol/biotin‐cap‐PE + TR‐DHPE (58/10/30/2 + 0.2 mol%) b), DPPC/DPPS/cholesterol/biotin‐cap‐PE + TR‐DHPE (58/10/30/2 + 0.2 mol%) c), DOPC/DOPS/DPPC/cholesterol/biotin‐cap‐PE + TR‐DHPE (23/10/35/30/2 + 0.2 mol%) d), DOPC/DPPC/DPPS/cholesterol/biotin‐cap‐PE + TR‐DHPE (33/25/10/30/2 + 0.2 mol%) e). The data were obtained from n >95 GUVs. Imaging was performed at 20 °C. Scale bars, 5 µm.

Without the formation of large pores, arginine‐rich CPPs can be internalized into PS‐containing GUVs, but at different rates for different phases of lipid membranes (**Figure** **4**). To determine which phase is more helpful for the penetration of R_9_, we prepared anionic L_d_ or L_o_ phase GUVs, which can be represented by DOPC/DOPS/cholesterol or DPPC/DPPS/cholesterol, respectively. To visualize and quantify the penetration of R_9_, we used FITC as a fluorophore conjugated to R_9_. We note that due to its anionic nature under the experimental pH, the physicochemical properties of the peptide can be altered, potentially affecting its ability to penetrate the membrane.^[^ ^43^, ^44^ ^]^ Into the L_d_ phase GUVs, the penetration of FITC‐R_9_ was extremely rapid even at 5 µm FITC‐R_9_, so that the fraction of R_9_‐internalized GUVs reached 100% in just 10 min (Figure 4a, empty red diamond); note that we extrapolated the FITC‐R_9_ concentrations inside GUVs from their fluorescence intensities using confocal fluorescence microscopy images, such that GUVs with >0.2 µm FITC‐R_9_ were regarded as the R_9_‐internalized GUVs (Figure S7, Supporting Information). On the other hand, the entry of FITC‐R_9_ into the L_o_ phase GUVs occurred less frequently than that into the L_d_ phase GUVs (empty black diamond). In particular, when comparing the quantities of FITC‐R_9_ between the two different GUVs, the L_d_ phase GUVs composed of unsaturated lipids (Figure 4b) exhibited a higher accumulation of FITC‐R_9_ in their lumens compared to the L_o_ phase GUVs composed of saturated lipids (Figure 4c), which is in good agreement with previous studies showing a rapid penetration of arginine‐rich CPPs through the membranes composed of unsaturated lipids.^[^ ^15^, ^16^ ^]^ Higher concentrations of FITC‐R_9_ accelerated its accumulation in both the L_d_ and L_o_ phase GUVs (Figure S8a,b, Supporting Information), and the absence of anionic lipids caused neither calcein leakage nor FITC‐R_9_ penetration even at high R_9_ concentrations (≈50 µm) (Figure S9, Supporting Information).

As we have previously demonstrated in planar membranes, the presence of arginine‐rich CPPs in phase‐separated GUVs can cause a further phase transition from L_o_ to L_d_ phase, facilitating their translocation across more fluidized membranes. Similar to the single‐phase GUVs, PS‐containing phase‐separated GUVs allowed consistent penetration of FITC‐R_9_, and the presence of anionic L_d_ domains was more effective for membrane penetration than that of anionic L_o_ domains; at 5 µm FITC‐R_9_, 97.1% DOPS‐containing GUVs were determined as R_9_‐internalized GUVs within 10 min (Figure 4a, solid red square), and the DPPS‐containing GUVs displayed 74.8% during the same period (solid black square). However, compared to the DPPS‐containing homogeneous GUVs (empty black diamond) with the same content of PS and cholesterol (10 and 30 mol%, respectively), the DPPS‐containing heterogeneous GUVs could accelerate the penetration of R_9_, attributed to the dissolution of L_o_ domains into L_d_ ones upon the phase‐preferential R_9_ binding. Lipid packing defects at the domain boundary of phase‐separated membranes may also have a supplementary effect on enhancing R_9_ internalization.^[^ ^45^ ^]^ Even the time‐dependent R_9_ accumulations in the DPPS‐containing heterogeneous GUVs (Figure 4e) were much larger than those in the DPPS‐containing homogeneous GUVs at 5 µm FITC‐R_9_ (Figure 4c). Similar trends were observed at higher R_9_ concentrations (Figure S8b–d, Supporting Information). Although the time‐dependent R_9_ accumulations were smaller in the DOPS‐containing heterogeneous GUVs (Figure 4d; Figure S8c, Supporting Information) than those in the DOPS‐containing homogeneous GUVs (Figure 4c; Figure S8a, Supporting Information), these would be due to the different area fraction of L_d_ domains relevant to the total amount of unsaturated lipids.

### 2.5 Dynamics of R_9_ Binding and Its Penetration into Phase‐Separated GUVs

**Figure 5:**
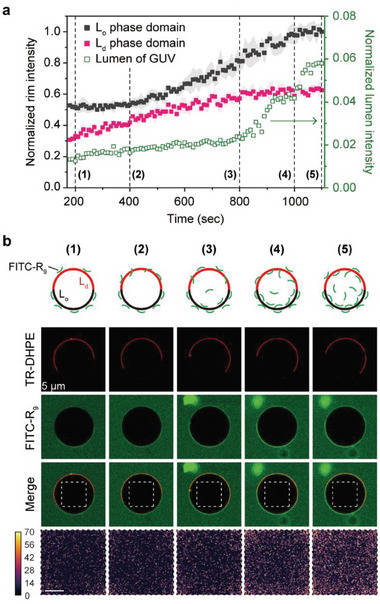
Real‐time tracking of FITC‐R_9_ entry into a DOPC/DPPC/DPPS/cholesterol GUV. a) Time‐dependent FITC‐R_9_ accumulations at the rim and in the lumen of the phase‐separated GUV. The FITC fluorescence intensities of L_o_ and L_d_ phase rims (solid black and red squares, respectively) were normalized to the final intensity of L_o_ phase rim. Data represent mean ± SD (n = 5). The intensities of the lumen (empty green square) were normalized to the bulk intensity outside the GUV. b) Confocal fluorescence microscopy images of the phase‐separated GUV at each time point. At (1), the binding of FITC‐R_9_ to the outer surface of the GUV reached equilibrium. Between (1) and (2), only the intensity of L_d_ phase rim increased, indicating that the FITC‐R_9_ would be continuously translocated through the L_d_ domain. From (2), the intensity of L_o_ phase rim began to increase as the FITC‐R_9_ in the lumen bound to the inner surface of the L_o_ domain. At (3), the intensity of L_d_ phase rim reached a plateau, but that of the lumen began to increase rapidly. At (4), the intensity of L_o_ phase rim also reached a plateau, but that of the lumen consistently increased from (4) to (5). The images in the last row are intensity mappings of the GUV lumen (white dashed squares in merged channels). Membrane composition: DOPC/DPPC/DPPS/cholesterol/biotin‐cap‐PE + TR‐DHPE (33/25/10/30/2 + 0.2 mol%). Imaging was performed at 20 °C. Scale bar, 5 µm.

To further understand the arginine‐rich CPP‐mediated lipid rearrangements and its active translocation across laterally heterogeneous membranes, we visualized the R_9_ binding and its penetration in real‐time in the coexistence of L_d_ and L_o_ phase domains (**Figure** **5**). Using FITC‐R_9_ (green) and TR‐DHPE (red), we readily assess peptide binding at L_d_ domains with strong red fluorescence and L_o_ domains with weak red fluorescence; the phase‐preferential R_9_ binding and subsequent R_9_ accumulation were evaluated with the intensity of FITC fluorescence at the rim of each phase and in the lumen of GUVs composed of DOPC/DPPC/DPPS/cholesterol (Figure 5a). In measuring the rim intensities, a straight line was drawn through three points: the L_o_ phase region, the GUV center, and the L_d_ phase region (Figure S10, Supporting Information). The first and second highest peaks in the TR‐DHPE intensity profile corresponded to the L_d_ and L_o_ phase regions, respectively. The FITC intensity at each peak represented the concentration of FITC‐R_9_ at each phase domain, and we obtained the average values from 5 distinct lines.

As soon as the DPPS‐containing heterogeneous GUVs were exposed to FITC‐R_9_, their outer surfaces were fully covered with the bound FITC‐R_9_, with a larger amount bound to the L_o_ domain than at the L_d_ domain (point (1) in Figure 5). For the following 200 s (from point (1) to (2)), the normalized intensity of L_o_ phase rim (solid black square) was maintained at ≈0.5, while that of L_d_ phase rim (solid red square) slowly increased, inferring that the FITC‐R_9_ would not pass through the L_o_ domain but through the L_d_ domain. However, at point (2), the intensity of L_o_ phase rim increased with the time delay; presumably, the FITC‐R_9_ that entered the GUV through the L_d_ domain would be bound to the inner surface of the L_o_ domain rather than being free in the lumen, as there was no significant increase of accumulated FITC‐R_9_ (empty green square). Nevertheless, a certain fraction of the peptide did not adhere to the inner membrane surface and instead remained within the lumen. At point (3), the intensity of L_d_ phase rim reached a plateau with no further increase, but that of GUV lumen began to increase rapidly, indicating continuous penetration of FITC‐R_9_ and its subsequent accumulation in the GUV. As the concentration of FITC‐R_9_ inside the GUV became sufficiently high, at point (4) the intensity of L_o_ phase rim also reached a plateau, suggesting that even the inner surface of the L_o_ domain would be completely occupied with the internalized FITC‐R_9_. However, the intensity of the GUV lumen consistently increased with FITC‐R_9_ accumulation over time (from point (4) to (5)).

The preferential penetration of R_9_ through the L_d_ domain suggests that when L_d_ and L_o_ domains coexist in phase‐separated membranes, translocation of arginine‐rich CPPs would favor the thinner and less rigid domains; in other words, the ordered domains have a higher concentration threshold for peptide penetration. Based on the previously reported phase diagrams,^[^ ^46^, ^47^, ^48^ ^]^ we estimated that with our membrane composition (10 mol% DPPS), the L_d_ and L_o_ phase domains would contain ≈7–13 mol% DPPS, respectively. Because of lower charge density, the L_d_ domain was less covered with the FITC‐R_9_ than the L_o_ domain, but the initial increase in the intensity of L_d_ phase rim validated that the membrane flexibility would be more crucial for the penetration of FITC‐R_9_. As the penetration of R_9_ across the less anionic L_d_ domains prevails over that across the more anionic L_o_ domains, it is evident that the R_9_‐induced domain dissolution from a more ordered state to a less ordered state would actively lead to their easier translocation across laterally heterogeneous membranes. This implies that the dissolution of the L_o_ phase into the L_d_ phase induced by R_9_ plays a substantial role in facilitating the penetration of the peptide.

## 3 Conclusion

**Figure 6:**
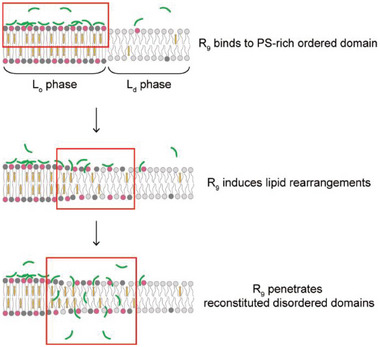
The potential mechanism for accelerating direct translocation of R_9_ into laterally heterogeneous membranes with membrane reconstitution and permeabilization. Cationic R_9_ binds to anionic L_o_ phase domains due to their strong electrostatic interactions. R_9_ binding actively induces lipid rearrangements, allowing phase transition from L_o_ to L_d_ phase for membrane fluidization. As a more loosely packed state, the reconstituted L_d_ phase domain becomes a route for accelerating the direct translocation of R_9_ across the phase‐separated membranes.

In exploring how arginine‐rich CPPs traverse laterally heterogeneous cell membranes, we directly visualized in real‐time the dynamic reorganization of membranes with coexisting L_d_ and L_o_ phases mediated by R_9_ binding and its penetration. By analyzing the fluorescence intensities of FITC‐R_9_ at the surface and in the lumen of phase‐separated GUVs, we observed that R_9_ translocated through the disordered domains rather than the ordered domains. This result is consistent with previous findings that the penetration of R_9_ was readily observed when the membrane was mechanically weak and deformable, but became rare when it was not.^[^ ^15^, ^45^, ^49^ ^]^ However, we also demonstrated that R_9_ eventually dissolved the L_o_ domains containing anionic lipids, suggesting that the arginine‐rich CPPs actively alter the spatial and temporal distribution of membrane components. Presumably, in the heterogeneous GUVs, additional R_9_ would lead to lateral expansion of ordered domains to form energetically unfavorable voids, and hydrocarbon chains of neighboring lipids would rearrange into a more disordered conformation to remove the free volumes in the bilayer.^[^ ^50^, ^51^ ^]^ Owing to the R_9_‐induced membrane reorganization_,_ the heterogeneous membranes would become fluidized, thereby facilitating subsequent translocation of R_9_ across otherwise highly ordered, impermeable membranes (**Figure** **6**).

The finding of this study, demonstrating that the dissolution of ordered membrane domains promotes the penetration of CPPs, is in accordance with established explanations underlying membrane permeability. Membrane thickness is an important factor in determining permeability across the membrane; the disordering of membrane lipids typically leads to a decrease in membrane thickness,^[^ ^52^ ^]^ subsequently lowering the energy barrier for peptide penetration.^[^ ^49^, ^53^ ^]^ In particular, the transition of the membrane from L_o_ to L_d_ phase dramatically improves the permeability of small molecules through the membrane,^[^ ^54^, ^55^ ^]^ which is consistent with our results with R_9_. Furthermore, an increase in the lipid bilayer fluidity makes the bilayer more susceptible to further membrane reconstructions, such as alterations in membrane curvature^[^ ^16^, ^32^, ^56^ ^]^ and redistribution of membrane components,^[^ ^13^, ^29^, ^32^, ^57^ ^]^ which have been proposed as a potential mechanism for CPP translocation.

In a cell, negatively charged lipids are not uniformly distributed throughout the plasma membrane. For instance, PS is predominantly located at the inner leaflet of the eukaryotic plasma membrane.^[^ ^58^ ^]^ This asymmetry is disrupted in cancer cells, as well as other pathological and apoptotic cells.^[^ ^59^, ^60^ ^]^ Consequently, PS has emerged as a promising target for anticancer therapeutics,^[^ ^61^ ^]^ including arginine‐rich CPPs.^[^ ^62^, ^63^ ^]^ Additionally, a considerable fraction of PS is found to co‐segregate with cholesterol to form distinct microdomains called lipid rafts,^[^ ^64^, ^65^, ^66^, ^67^ ^]^ and the lipid rafts are generally accepted to exist as L_o_ domains.^[^ ^68^, ^69^ ^]^ Accordingly, it is typically regarded that penetration of CPPs would be significantly challenging through the highly ordered lipid rafts; previous studies have reported that increasing the structural order of membranes, particularly with increasing cholesterol content, suppresses the translocation of CPPs.^[^ ^15^, ^33^, ^34^ ^]^ To increase the accumulation of CPPs within cells or vesicles, perturbation of lipid packing, such as cholesterol extraction and the addition of membrane‐disrupting molecules, has been suggested.^[^ ^57^, ^70^, ^71^, ^72^ ^]^ In this regard, our current findings expand previous works by demonstrating that CPPs themselves can locally loosen the packing of pathological cell membranes through lateral membrane reorganization prior to their active translocation. We believe that this complements existing model membrane studies that have assessed the translocation of CPPs only with overall membrane properties,^[^ ^73^ ^]^ by elucidating how locally concentrated CPPs reconstitute heterogeneous lipid membranes, thereby providing insight into highly effective drug delivery.

## 4 Experimental Section

### Materials

1,2‐dioleoyl‐sn‐glycero‐3‐phosphocholine (DOPC), 1,2‐dipalmitoyl‐sn‐glycero‐3‐phosphocholine (DPPC), 1,2‐dioleoyl‐sn‐glycero‐3‐phospho‐L‐serine (DOPS), 1,2‐dipalmitoyl‐sn‐glycero‐3‐phospho‐L‐serine (DPPS), 1,2‐dimyristoyl‐sn‐glycero‐3‐phosphocholine (DMPC), 1,2‐dimyristoyl‐sn‐glycero‐3‐phospho‐(1′‐rac‐glycerol) (DMPG), 1,2‐dioleoyl‐sn‐glycero‐3‐phosphoethanolamine‐N‐(cap biotinyl) (biotin‐cap‐PE), and cholesterol were purchased from Avanti Polar Lipids. Texas Red 1,2‐dihexadecanoyl‐sn‐glycero‐3‐phosphoethanolamine (Texas Red‐DHPE) was purchased from Thermo Fischer Scientific. Other chemicals, including hexadecane, silicone oil AR20, chloroform, ethanol, sodium chloride (NaCl), sodium tetraborate, 4‐2(2‐hydroxymethyl)−1‐piparazineethanesulfonic acid (HEPES), 1‐dodecanethiol, poly‐L‐lysine (PLL) hydrobromide, biotinylated bovine serum albumin (biotin‐BSA), streptavidin, glucose, calcein, methyl‐β‐cyclodextrin (MβCD), Fmoc‐protected arginine, 1‐Hydroxybenzotriazole hydrate (HOBT), N,N,N′,N′‐Tetramethyl‐O‐(1H‐benzotriazol‐1‐yl)uronium hexafluorophosphate (HBTU), Fmoc‐Arg(pbf)─OH, dimethylformamide (DMF), N,N‐diisopropylcarbodiimide (DIC), trifluoroacetic acid (TFA), triisopropylsilane (TIS), 3‐Aminobutanoic acid (Abu), and 5,6‐carboxyfluorescein (FITC) were obtained from Sigma Aldrich. Amine‐reactive polyethylene glycol (PEG), mPEG–succinimidyl valerate, (MW 5000), was purchased from Laysan Bio. Rink amide MBHA resin (0.45 mmol g^−1^) was purchased from Bead Tech. TAT (47‐57) peptide (HIV‐1 TAT) was purchased from AnaSpec. All the lipids were dissolved in chloroform or the chloroform/methanol (2:1 v/v), and stored at −20 °C. NaCl and HEPES were dissolved in distilled water to make the aqueous buffer containing 100 mm NaCl and 10 mm HEPES, followed by titration to pH 7.4 with 1 m NaOH solution, and stored at 4 °C.

### Peptide Synthesis

All peptides were synthesized with the solid‐phase peptide synthesis method. Rink amide MBHA resins (100 mg, 45 µmoL) were swollen with DMF (2 mL) in a 5 mL fritted syringe overnight. The Fmoc protecting group was removed by treatment with 20% piperidine in DMF (2 × 10 min). On the resin, arginine residues were added by a repeated standard route. In detail, for amino‐functionalized resin conjugation, Fmoc‐Arg(pbf)─OH (5 equiv.) were treated with 1 m HOBT (5 equiv.), 1 m HBTU (5 equiv.), and 1 m DIC (10 equiv.) in DMF. After shaking for 2 h, the reaction was finished and mixture was drained out and the resin was washed with DMF (7×). Unless noted, this washing step was repeated at each reaction step. This process was repeated until desired sequences of nona‐arginine (R_9_) were obtained. For cleavage, the beads were treated with 1 mL of a cleavage cocktail (95% TFA, 2.5% TIS, and 2.5% distilled water) for 2 h. To synthesize FITC‐labeled R_9_ (FITC‐R_9_), the N‐terminus of R_9_ was coupled with Abu linker by standard Fmoc chemistry. After deprotection, the amine group of the Abu was coupled with FITC (10 equiv.) using the peptide coupling chemistry. For cleavage, the beads were treated with 1 mL of a cleavage cocktail (95% TFA, 2.5% TIS, and 2.5% distilled water) for 2 h.

After reactions were completed, the crude products were purified by high performance liquid chromatography (HPLC), and their purity and identity were analyzed by liquid chromatography‐mass spectrometry (LC‐MS) and matrix‐assisted laser desorption ionization‐time of flight mass spectrometry (MALDI‐TOF MS). Analytical HPLC and LC‐MS characterization were performed on an Agilent system with a C18 reversed‐phase HPLC column (Agilent, 3.5 µm, 4.6 mm × 150 mm). A gradient elution of 10%–100% B in 7 min (keep 100% B till 13 min) was used at a flow rate of 0.7 mL mi^−1^n (solvent A: distilled water, 0.01% TFA; solvent B: acetonitrile, 0.01% TFA). With a C18 reversed‐phase column (Agilent, 5 µm, 21.2 mm × 150 mm) changing solvent composition with a linear gradient of 100% solvent A in 5 min followed by 100% solvent B in 65 min. MALDI‐TOF MS was performed on 4700 Proteomics Analyzer (Applied Biosystems) and Autoflex speed LRF (Bruker) using α‐cyano‐4‐hydroxycinnamic acid and 2,5‐dihydroxybenzoic acid as matrices. Small aliquots of the obtained R_9_ and FITC‐R_9_ were flash‐frozen using liquid nitrogen and stored at −20 °C. Peptides for long‐term storage were freeze‐dried.

### Preparation of Planar Lipid Membrane Imaging

The imaging chamber was assembled with a bottom coverslip and side slide glasses. PLL–PEG was then used to passivate the glass coverslips. PLL‐PEG was prepared by conjugation of amine‐reactive PEG with PLL at a molar ratio of 1:5. The reaction was performed in 50 mm sodium tetraborate pH 8.5 solution. The mixture was continuously stirred overnight at room temperature, buffer exchanged into 10 mm HEPES, 100 mm NaCl, pH 7.4 using a D‐Tube Dialyzer (MWCO 6–8 kDa, Novagen), and stored at 4 °C. The 200 µL of 0.5% PLL‐PEG solution was added to each imaging chamber. After 20 min of incubation, the chamber was rinsed with 10 mm HEPES, 100 NaCl, and pH 7.4 buffer.

### Freestanding Planar Lipid Membranes

Freestanding planar lipid membranes were prepared using an ultra‐stable freestanding planar lipid membrane array, as described in previous studies.^[^ ^37^, ^38^ ^]^ First, the lipids dissolved in chloroform were mixed in a glass vial in the desired molar ratio and dried under a gentle N_2_ stream. The dried lipids were dissolved in a mixture of hexadecane and silicone oil (1:1 v/v) to obtain a lipid oil solution with a total lipid concentration of 5 mm. Each oil was filtered through a 0.2 µm syringe filter (Whatman) to remove impurities before use. The lipid oil solution was sonicated for 1 h and used for experiments within 5 h.

A total of 1 mL of the aqueous buffer was injected into the PLL‐PEG passivated imaging chamber, and 2 µL of lipid oil solution was gently dropped and spread on the air–aqueous buffer interface. ≈3 min after dropping the lipid oil solution, a hexagonal TEM grid (G200HEX, G150HEX, G100HEX, Gilder Grids) treated with 1‐dodecanethiol for hydrophobic coating was gently placed on the air‐oil interface using tweezers. For the next 2 min, the hydrophobic surface of the TEM grid was wetted with the lipid oil solution, and the holes of the grid were filled with thin oil films. The grid was submerged into the aqueous buffer using a syringe needle to position it on the PLL‐PEG‐coated glass surface. As the oil drained, the thickness of the oil film decreased sufficiently for zipping of the two lipid monolayers, which resulted in the formation of the planar freestanding lipid bilayers. The lipid membrane array was incubated at 45 °C for at least 30 min and cooled to 24 °C. ≈1 h after cooling, a cholesterol‐methyl‐β‐cyclodextrin solution (4 mm, 20:1 mol%) was injected into the aqueous buffer at a concentration of 60 mm to increase the cholesterol concentration in the lipid membranes to ≈30 mol%. After ≈1 h of incubation, the prepared R_9_ or FITC‐R_9_ solution was injected into the aqueous solution to the desired concentration. Experiments were done at 24 °C. The lipid membranes were directly visualized using an inverted fluorescence microscopy (IX73, Olympus) with an iXon EMCCD camera (X‐6880, Andor Solis). Images were analyzed using the open‐source program, Fiji.^[^ ^74^ ^]^

### GUV Preparation

The lipids dissolved in chloroform were mixed to a desired ratio at a total concentration of 1 mg mL^−1^. For all GUV experiments, 2 mol% of biotin‐cap‐PE was included for vesicle tethering on the glass surface. GUVs were prepared based on the electroformation method.^[^ ^75^ ^]^ Briefly, the lipid mixture in chloroform was spread on indium‐tin‐oxide coated glass slides (30 Ω, Omniscience) and dried under vacuum for at least 2 h. Electroporation was performed at 55 °C in 200 mOsm L^−1^ glucose solution or 200 mOsm L^−1^ glucose solution with 10 µm calcein. The voltage was increased from 50 to 1400 mVpp for the first 30 min at 10 Hz, and then held at 1400 mVpp for 120 min. For the last 30 min, voltage and frequency were adjusted to 2200 mVpp and 5 Hz, respectively.

### Coating of Glass Surface for GUV Tethering

GUVs were tethered on a glass coverslip using the strong interaction between biotin and streptavidin. The imaging chamber was assembled with a coverslip and 2 mm thick silicon gaskets with 3 mm diameter holes. Prior to chamber assembly, gaskets, and coverslips were cleaned with 2 vol% Micro‐90 concentrated cleaning solution (International Products Corporation), rinsed thoroughly with water, and dried under a nitrogen stream. 10 µL of 1 mg mL^−1^ biotin‐BSA solution was added to the chamber and incubated for at least 30 min. Excess biotin‐BSA was washed with 10 mm HEPES, 100 mm NaCl, and pH 7.4 buffer by gentle pipetting. Next, 0.2 mg mL^−1^ streptavidin was added to the chamber, incubated for at least 30 min, and then rinsed with the same buffer.

### Peptide Internalization Assay and Fluorescence Leakage Assay with GUVs

GUVs were observed using a confocal laser scanning microscope (LSM880, Carl Zeiss) in the KAIST Analysis Center for Research Advancement (KARA). To investigate R_9_‐induced pore formation of GUVs, calcein‐encapsulated GUVs were tethered on a glass coverslip and R_9_ solution was added to the desired concentration. To investigate the internalization of fluorescence‐labeled peptides into GUVs, calcein‐free GUVs were tethered on a glass coverslip and FITC‐R_9_ solution was added to the desired concentration. GUVs were exposed to the laser only when imaging to minimize the photobleaching of the fluorescence probes. Imaging was done at 20 °C and images were analyzed using the open source program, Fiji.^[^ ^74^ ^]^

### Statistical Analysis

The normalized area and normalized calcein fluorescence intensities inside the GUVs values were obtained by normalizing to the initial values before peptide addition. The FITC fluorescence intensities at GUV rims were normalized to the final intensity of each rim, and the FITC intensities of GUV lumen were normalized to the bulk intensity outside the GUV. The data shown in the graphs were described as mean ± SD of at least two independent experiments. The number of samples for each analysis was specified in the figure legends.

## Conflict of Interest

The authors declare no conflict of interest.

## Supporting information

Supporting Information

Supplemental Movie 1

## Data Availability

The data that support the findings of this study are available from the corresponding author upon reasonable request.
