# Supplementary material for: Arginine‐Rich Cell‐Penetrating Peptides Induce Lipid Rearrangements for Their Active Translocation across Laterally Heterogeneous Membranes
Source: Adv Sci (Weinh). 2024 Jun 26;11(32):2404563. doi: 10.1002/advs.202404563 (PMC11348069; doi:10.1002/advs.202404563)
Supplement: Supplementary file 1 — Supporting Information [file ADVS-11-2404563-s002.docx]

Supporting Information

Arginine-Rich Cell-Penetrating Peptides Induce Lipid Rearrangements for Their Active Translocation across Laterally Heterogeneous Membranes

Sujin Park, Jinmin Kim, Seung Soo Oh*, and Siyoung Q. Choi*


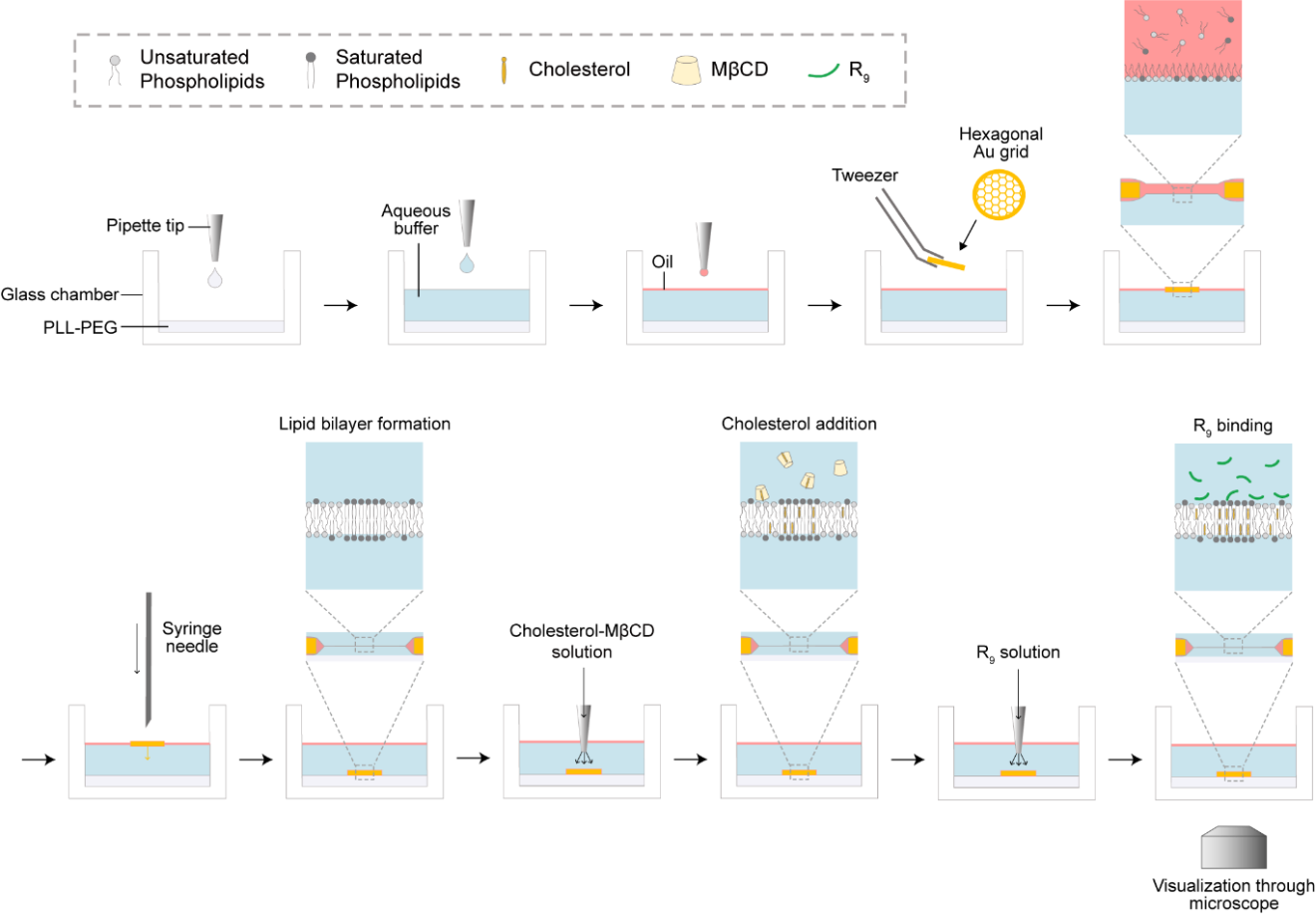


Figure S1. Schematic of experimental procedures for the formation of freestanding planar lipid membranes. MβCD = methyl-β-cyclodextrin, R_9_ = nona-arginine, TEM= transmission electron microscopy


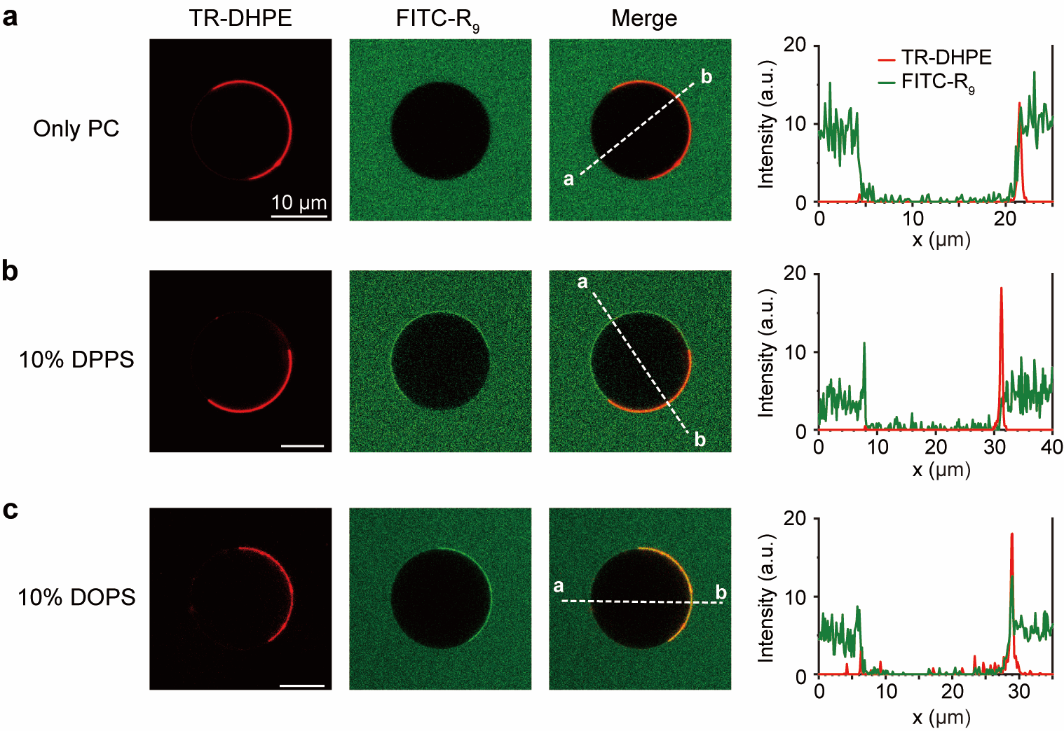


Figure S2. Interaction of R_9_ with PS-rich membrane phases. Confocal images of GUVs after addition of 30 μM FITC-R_9_. Graphs on the right are the fluorescence intensity profiles along the dotted line (from a to b) in the merged channel. Membrane compositions: DOPC/DPPC/cholesterol/biotin-cap-PE (33/35/30/2) + 0.2 mol% TR-DHPE (a), DOPC/DPPC/DPPS/cholesterol (33/25/10/30) + 0.2 mol% TR-DHPE (b), DOPC/DOPS/DPPC/cholesterol (23/10/35/30) + 0.2 mol% TR-DHPE (c). Imaging was done at 20°C, after 5 to 10 min after peptide addition. Scale bars, 10 μm.


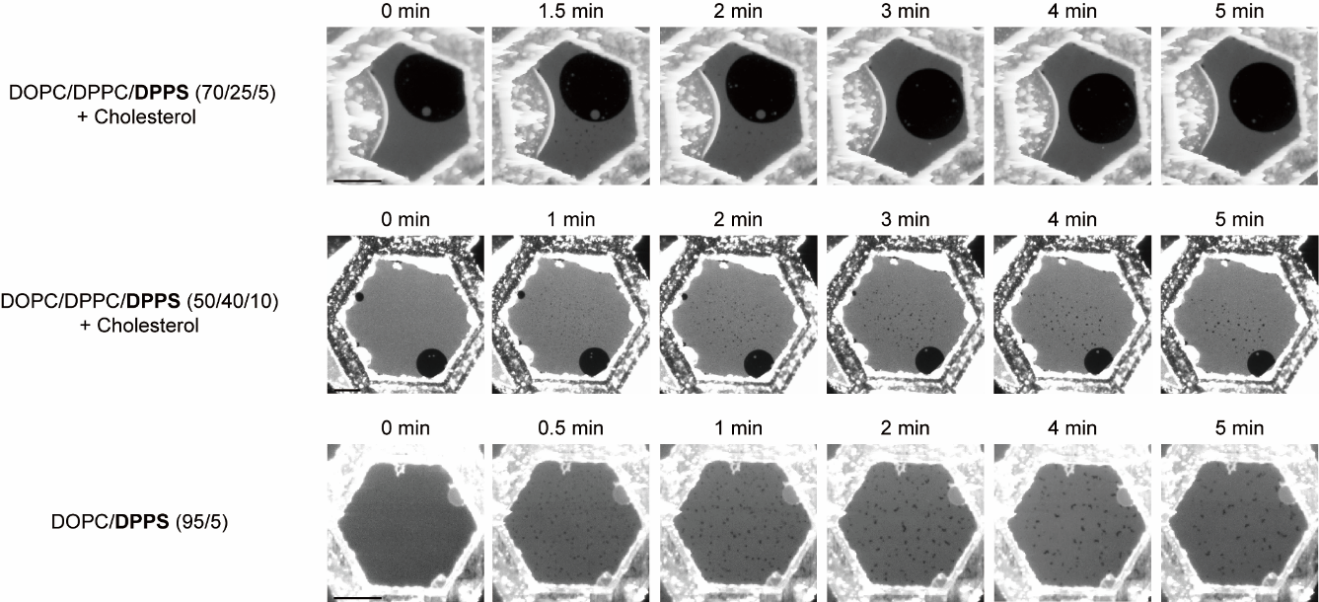


Figure S3. Formation of PS-rich domains upon addition of R_9_. Representative fluorescence images of membranes after the addition of R_9_ at 50 μM. The membranes were formed using lipid oil mixtures in the ratios indicated on the left with the addition of 0.5 mol% TR-DHPE, and cholesterol was added to the membranes via MβCD. Small L_o_ domains appeared at L_d_ phase within 1 min after injecting R_9_ solution. The elapsed time after injection of R_9_ solution is indicated above each image. Imaging was done at 24°C. Scale bars, 50 μm.


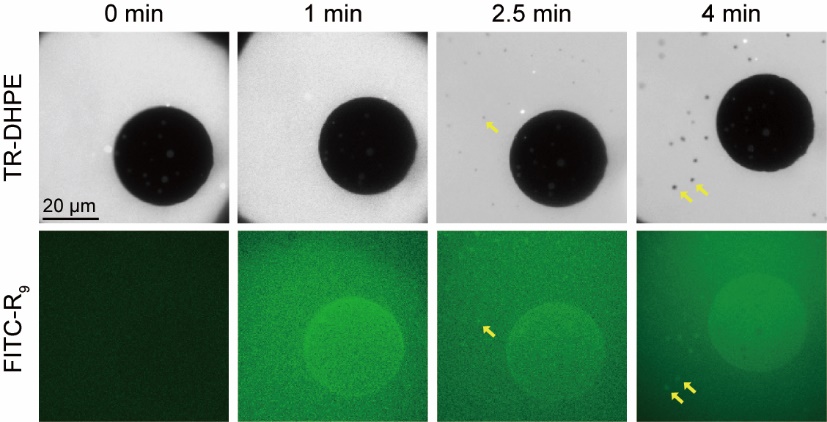


Figure S4. Binding of FITC-R_9_ to the PS-rich domains. Fluorescence images of membranes after the addition of FITC-R_9_ at 3 μM. The membranes were formed using a lipid oil mixture of DOPC/DPPC/DPPS (60/30/10) + 0.5 mol% TR-DHPE, and cholesterol was added to the membranes via MβCD. As indicated by the yellow arrows, small L_o_ domains appeared at L_d_ phase are observed bright in FITC-R_9_ fluorescence. The elapsed time after injection of R_9_ solution is indicated above each image. Imaging was done at 24°C. Scale bar, 20 μm.

**
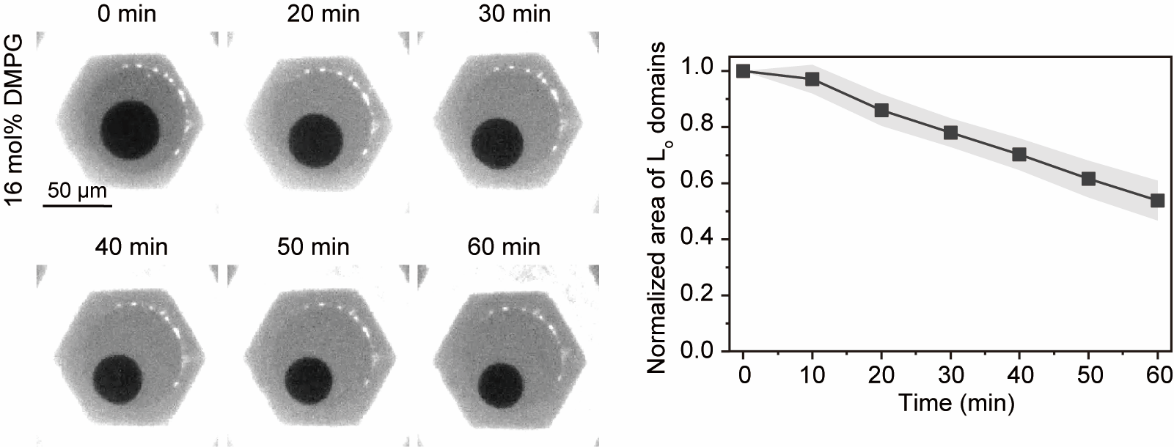
**

Figure S5. Visualization of DOPC/DMPC/DMPG/cholesterol membrane upon addition of HIV-1 TAT. Left: Representative fluorescence images of DOPC/DMPC/DMPG/cholesterol membrane after the addition of HIV-1 TAT at 4 μM. The elapsed time after injection of TAT solution is indicated above each image. Right: Normalized area of L_o_ domain over time. Normalized area (A_R_) was defined as A_R_= A/A_0_, where A and A_0_ respectively indicate the area of the L_o_ domain at each time point and the initial area of the L_o_ domain before injecting HIV-1 TAT solution. The membranes were formed using a lipid oil mixture of DOPC/DMPC/DMPG+ TR-DHPE (50/34/16 + 0.5 mol%) and cholesterol was added to the membranes via MβCD. Scale bar, 50 μm.


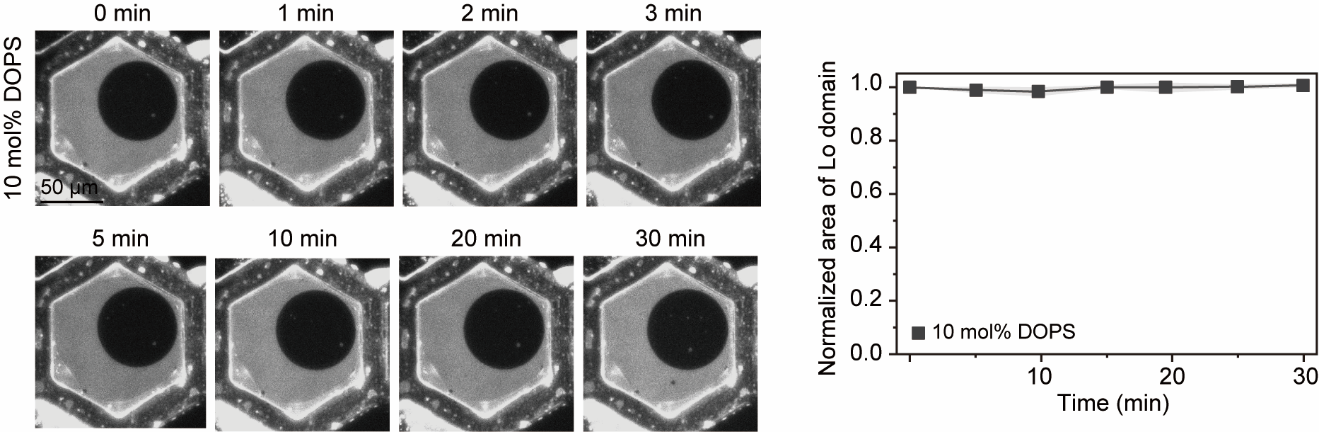


Figure S6. Visualization of DOPC/DOPS/DPPC/cholesterol membrane upon addition of R_9_. Left: Representative fluorescence images of DOPC/DOPS/DPPC/cholesterol membrane after the addition of R_9_ at 50 μM. The elapsed time after injection of R_9_ solution is indicated above each image. Right: Normalized area of L_o_ domain over time. Normalized area (A_R_) was defined as A_R_= A/A_0_, where A and A_0_ respectively indicate the area of the L_o_ domain at each time point and the initial area of the L_o_ domain before injecting R_9_ solution. The membranes were formed using a lipid oil mixture of DOPC/DOPS/DPPC + TR-DHPE (50/10/40 + 0.5 mol%) and cholesterol was added to the membranes via MβCD. Scale bar, 50 μm.


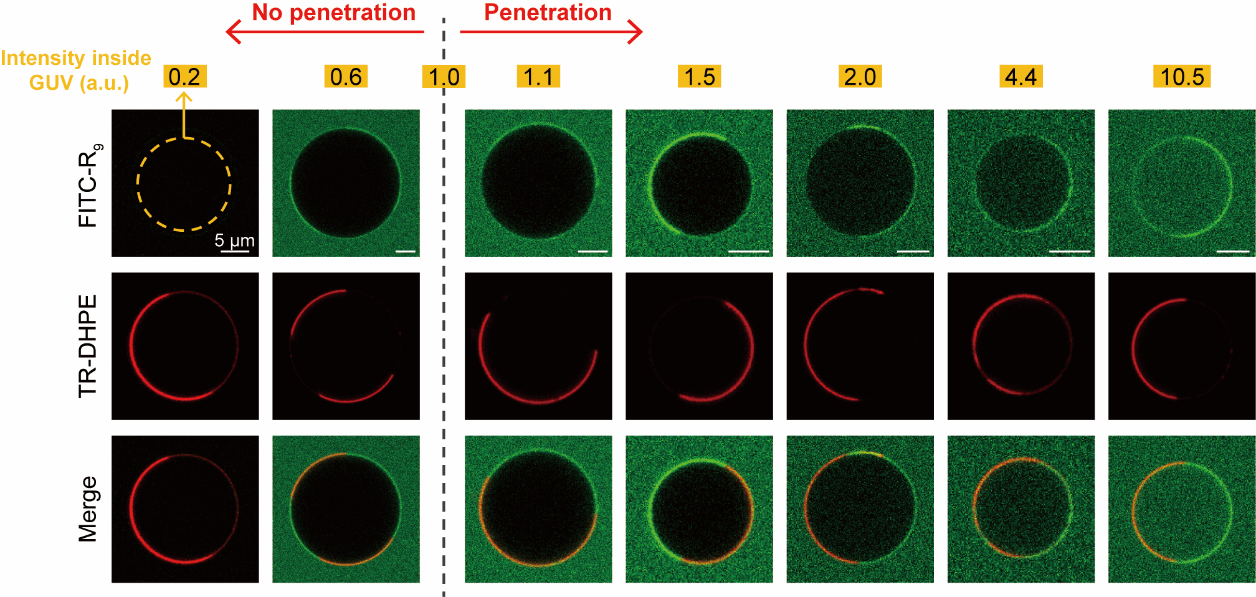


Figure S7. Criteria for determining whether FITC-R_9_ has entered the GUV interior. The penetration of R_9_ was determined by measuring the fluorescence intensity inside the GUV. If the lumen intensity was less than 1, where the concentration of FITC-R_9_ is approximately 0.2 μM, it was determined that there was no penetration, and if it was greater than 1, it was determined that there was penetration.


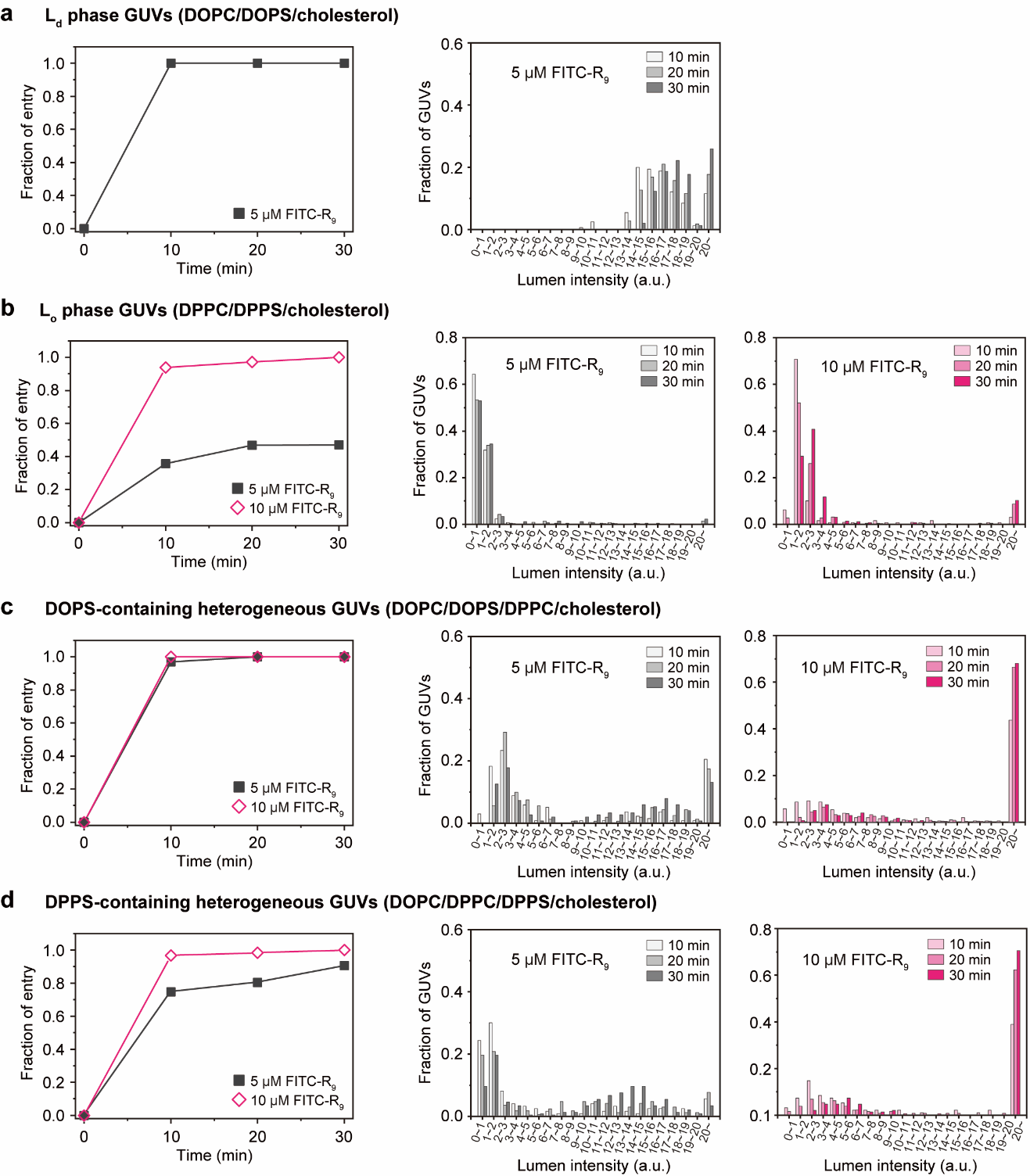


Figure S8. Entry of FITC-R_9_ into GUVs of different compositions. Fraction of entry was analyzed with 4 different mem-brane compositions at two different FITC-R_9_ concentrations. L_d_ phase GUVs (a), L_o_ phase GUVs (b), DOPS-containing heterogeneous GUVs (c), and DPPS-containing heterogeneous GUVs (d), were treated with FITC-R_9_ at 5 μM (black) and 10 μM (pink), respectively. The histograms on the right show the distribution of FITC fluorescence intensity inside GUVs after exposure to FITC-R_9_ for 10, 20, and 30 minutes. Membrane compositions: DOPC/DOPS/cholesterol/biotin-cap-PE + TR-DHPE (58/10/30/2 + 0.2 mol%) (a), DPPC/DPPS/cholesterol/biotin-cap-PE + TR-DHPE (58/10/30/2 + 0.2 mol%) (b), DOPC/DOPS/DPPC/cholesterol/biotin-cap-PE + TR-DHPE (23/10/35/30/2 + 0.2 mol%) (c), DOPC/DPPC/DPPS/cholesterol/biotin-cap-PE + TR-DHPE (33/25/10/30/2 + 0.2 mol%) (d).


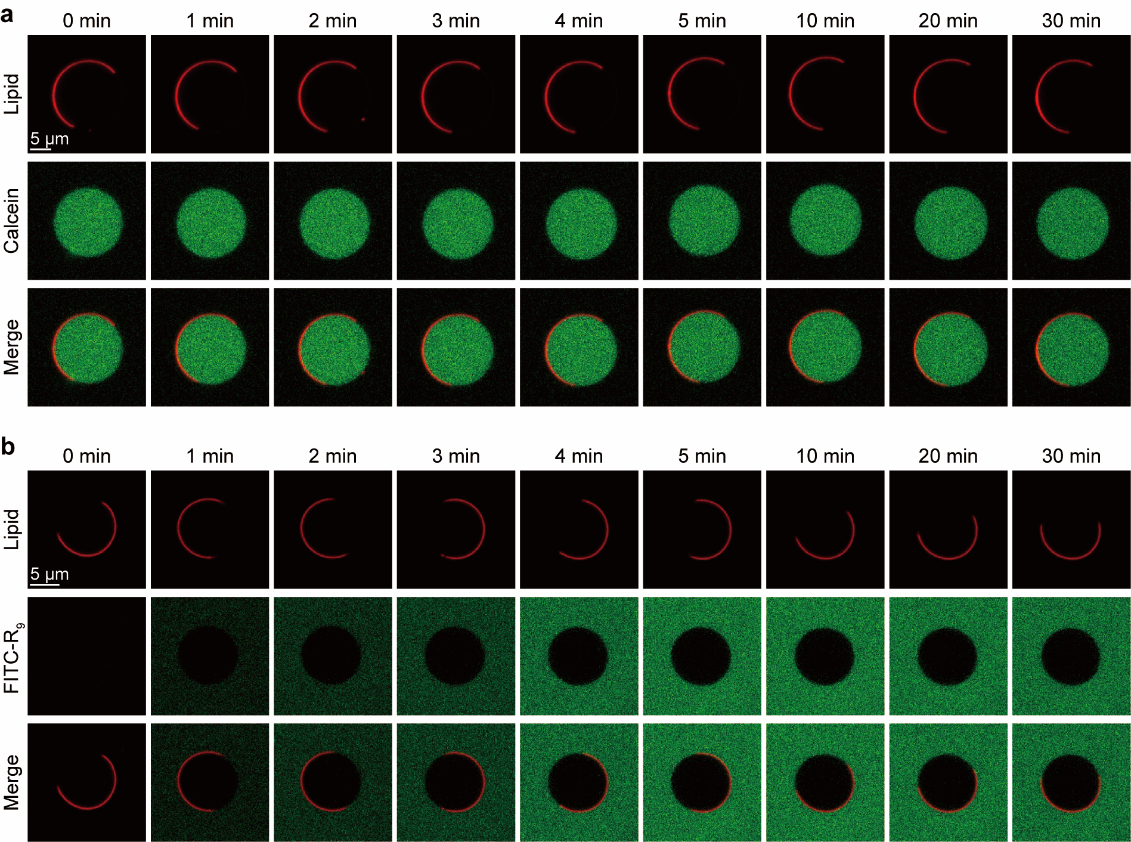


Figure S9. Leakage of calcein and entry of FITC-R_9_ with DOPC/DPPC/cholesterol GUVs. a, Confocal images of DOPC/DPPC/cholesterol GUVs containing calcein after addition of 50 μM R_9_. b, Confocal images of DOPC/DPPC/cholesterol GUVs after addition of 50 μM FITC-R_9_. Membrane composition: DOPC/DPPC/cholesterol/biotin-cap-PE (33/35/30/2) + 0.2 mol% TR-DHPE. The elapsed time after injection of the peptide solution is indicated above each image. Imaging was done at 20°C. Scale bars, 5 μm.


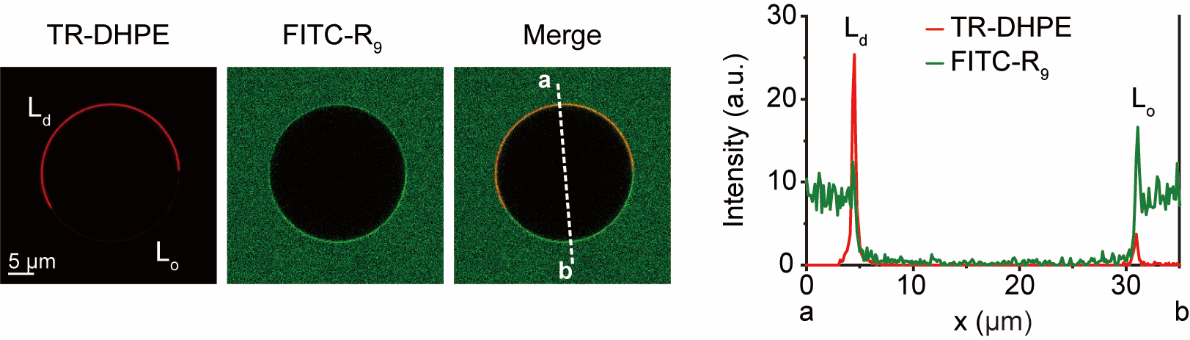


Figure S10. Measurement of rim intensities using GUVs with coexisting L_o_/L_d_ phases. Left: Confocal images of DOPC/DPPC/DPPS/cholesterol GUVs. In the TR-DHPE channel, L_d_ domains look bright while L_o_ domains look weakly fluorescent. A line was drawn through the center of the GUV and each phase region. Right: Graphs are the fluorescence intensity profiles along the dotted line (from a to b) in the merged channel. Each peak represents the intensity of TR-DHPE (red) and FITC-R_9_ (green) of the GUV rim at the L_d_ and L_o_ phase domains, respectively. Membrane composition: DOPC/DPPC/cholesterol/biotin-cap-PE (33/35/30/2) + 0.2 mol% TR-DHPE. Imaging was done at 20°C. Scale bars, 5 μm.

Movie S1. R_9_-induced remodeling of the lipid membrane shown in Figure 2b. At the beginning of the movie, R_9_ solution was injected at a concentration of 50 μM. The membranes were formed using a lipid oil mixture of DOPC/DPPC/DPPS + TR-DHPE (60/30/10 + 0.5 mol%), and cholesterol was added to the membranes via MβCD. The movie is speeded up 100x. Imaging was done at 24°C. Scale bar, 100 μm.
